# Overcoming racial disparities in cancer clinical trial enrollment of Asians and Native Hawaiians

**DOI:** 10.1016/j.conctc.2022.100933

**Published:** 2022-05-27

**Authors:** Jared D. Acoba, Ken Sumida, Jeffrey Berenberg

**Affiliations:** a University of Hawaii Cancer Center, Honolulu, HI, USA; b John A. Burns School of Medicine, Honolulu, HI, USA; c Tripler Army Medical Center, Honolulu, HI, USA

**Keywords:** Clinical trial, Race, Disparities, Cancer, Hawaiian, Asian

## Abstract

**Background:**

Asians and Native Hawaiians are two of the fastest growing minority populations in the United States, however these racial minority groups are severely underrepresented in clinical trials. This study looks at cancer clinical trial accrual among Asians and Native Hawaiians in a community-based network with a mission of increasing minority accrual to studies.

**Methods:**

The University of Hawaii Cancer Center (UHCC) network enrolls patients to treatment and non-treatment cancer studies. Enrollment on studies opened between 2009 and 2013 were obtained from UHCC's clinical trial management system. Incidence of cancer by race was acquired from the Hawaii Tumor Registry. Enrollment fractions were compared for the most common races in the state: White, Asian (specifically Chinese, Filipino, Japanese), and Native Hawaiian.

**Results:**

Whites comprised the largest proportion of cancer patients and participants in trials. Asians and Native Hawaiians were enrolled into cancer clinical trials at the same or higher enrollment fraction compared to Whites. Chinese, Japanese, and Native Hawaiian patients participated in treatment trials significantly more often than Whites (p < 0.05). Similarly, Chinese and Native Hawaiians enrolled in non-treatment trials at a significantly higher rate compared to Whites (p < 0.05).

**Conclusions:**

The UHCC network has instituted many strategies to increase minority accrual that have likely led to Asian and Native Hawaiian patients participating in studies at least as often as White patients. The strategies implemented at UHCC may benefit similar communities with a high number of minority cancer patients.

Clinical trials represent the standard of care for oncology treatment, yet only 2–3% of adult cancer patients participate in a clinical trial [1,2]. Just as concerning, there is a significant racial disparity in clinical trial participation. Racial minorities make up 36% of the total US population, but only 15–20% of clinical trial participants [3]. Murthy et al. demonstrated that enrollment of minorities on NCI sponsored trials was significantly lower than enrollment of Whites [4]. A more recent study demonstrated that rates of minority enrollment actually decreased from 2003 to 2016 [5].

Asians and Native Hawaiians are two of the fastest growing populations based on 2020 US Census data [6,7]. Similar to other racial minorities, Asians and Native Hawaiians have been underrepresented in cancer clinical trials. While the percent of Asians accrued to treatment studies increased between the periods of 1990–2000 vs 2001–2010, accrual rates remained extremely low representing only 0.04% and 3.3% of the total enrollment respectively [8]. Analyses of patients enrolled in US Food and Drug Administration trials of prostate cancer [9] and ovarian and breast cancer [10] showed that Asians and Native Hawaiians were severely underrepresented. In the state of Hawaii, racial discrepancies in cancer prevention and treatment trial participation have been demonstrated. Bantum et al. reported that Native Hawaiians made up a lower proportion of trial patients compared to Whites and Asians, and Native Hawaiian men were specifically accrued at a low rate [11]. While the rate of Native Hawaiians in clinical trials may be increasing [12], the total number of trial participants remains low.

A racially diverse study population is necessary to ensure that clinical trial findings are generalizable [3] to all cancer patients. The inclusion of minorities allows for the identification of racial differences in: molecular subtypes of tumors, quality of life outcomes, and treatment preferences [13]. More importantly, the low rate of minority accrual to cancer clinical trials raises a question of equal access to novel therapies and concern for a lack of healthcare equity [14].

Studies on the barriers to Asian and Native Hawaiian participation in clinical trials can be categorized as: patient factors, provider factors, and health system and societal factors. Patient factors such as the fear and mistrust of the healthcare system and clinical trials are expressed by minority patients including Asians and Native Hawaiians [12,[15], [16], [17], [18]]. A provider centered barrier to clinical trial accrual is the perception that minority patients are challenging to work with and viewed as poor study candidates who will decline participation [19]. Health system and societal factors that are unequally faced by minority patients include low socioeconomic status, lack of transportation, increased challenges obtaining childcare, and less flexible work schedules [16]. Institutions and programs that focus on the enrollment of minorities to cancer clinical trials have been able to overcome some of these barriers, however accrual rates remain low [18,20].

Our primary objective was to analyze the cancer clinical trial enrollment at a center that focuses on the enrollment of minority patients, and in particular Asians and Native Hawaiians.

## 1 Methods

### 1.1 Cancer Center and inclusion criteria

The University of Hawaii Cancer Center (UHCC) enrolls patients to various types of cancer studies: treatment, symptom control, screening and prevention, and cancer care delivery trials. Patients are enrolled through a network of clinics and hospitals across the state of Hawaii including Hawaii Cancer Care, Hawaii Pacific Health, Hawaii Oncology, Kuakini Medical Center, the Cancer Center of Hawaii, the Queen's Medical Center, and Tripler Army Medical Center. The UHCC network treats 70% of all cancer patients in the state. For purposes of this study, analysis was restricted to trials that were open between 2009 and 2013. Only studies that enrolled adults (age ≥18yo) were included. Studies for which enrollment was restricted to one site within the UHCC network and single patient use protocols (compassionate use of a novel medication) were excluded from the analysis.

### 1.2 Data collection and measurement

Clinical trial data were acquired through UHCC's clinical trial management system, OnCore, to monitor and track clinical trial activities. Patient demographic data and all clinical trial accrual data are captured through OnCore. Trials were designated as “Treatment Trials” if they fit the definition used by NCI-designated Cancer Centers (Cancer Center Support Guide V3.1.1). Histologic subtype and race and age of participants were captured through OnCore. Trial participants self-identified their primary race. The most common races in Hawaii were included for analysis: White, Asian (specifically Chinese, Filipino, Japanese), and Native Hawaiian.

Data on statewide cancer incidence are publicly available through the Hawaii Tumor Registry (HTR). HTR collects data on all cancer patients in the state of Hawaii as a participant in the National Cancer Institute Surveillance, Epidemiology, and End Results Program. Data are available on cancer diagnoses by histology and subdivided by race. For the tumor registry, patients similarly self-identified their primary race.

### 1.3 Outcomes

The main outcome of interest was the enrollment fraction which was defined as the number of trial enrollees divided by the recorded cancer cases in each subgroup [4,5].

### 1.4 Statistical methods

Nonparametric descriptive statistics were used to evaluate enrollment fractions by race. A p < 0.05 was considered statistically significant. Statistical analyses were performed with SPSS version 27.0 (IBM Corp).

### 1.5 Ethics

Approval for this study was granted by the 10.13039/100008782University of Hawaii Institutional Review Board.

## 2 Results

From 2008 to 2012, 32,360 people were diagnosed with cancer in Hawaii. Analysis was restricted to 29,982 cancer patients in the five largest racial groups: 10,604 (35%) Whites, 7934 (27%) Japanese, 4858 (16%) Hawaiian. 4717 (16%) Filipinos, and 1869 (6%) Chinese (Table 1). Other racial groups, such as Koreans, comprise less than 2% of the total number of cancer patients in the state and were excluded from the analysis. Breast and prostate cancer were the most common cancer diagnoses.

**Table 1:** Total number of patients diagnosed with cancer by race, 2008–2012.

	Race, n (%)				
**Histologic subtype**	Chinese	Filipino	Hawaiian	Japanese	White
Breast	289 (6)	716 (15)	981 (20)	1425 (29)	1450 (30)
Colon	207 (6)	594 (16)	493 (14)	1145 (32)	924 (26)
Lung	255 (7)	633 (17)	649 (17)	890 (23)	1088 (28)
Prostate	244 (6)	656 (17)	424 (11)	973 (25)	1263 (33)
Uterine	70 (6)	169 (14)	233 (20)	290 (25)	261 (22)
Other	804 (6)	1949 (14)	2078 (15)	3211 (24)	5618 (41)
**Total**	**1869 (6)**	**4717 (16)**	**4858 (16)**	**7934 (27)**	**10604 (35)**

A total of 1909 patients enrolled in cancer clinical trials from 2009 to 2013. Of these 1515 patients belonged to one of the five racial groups included in this study. 681 patients were enrolled on treatment trials and 834 on non-treatment trials. Japanese patients made up the largest proportion of participants on treatment trials, and Whites made up the highest proportion of patients accrued to non-treatment studies (Table 2).

**Table 2:** Clinical trial enrollment by race, 2009–2013.

	Race, n (%)				
**Trial Type**	Chinese	Filipino	Hawaiian	Japanese	White
Treatment	47 (7)	87 (13)	157 (23)	205 (30)	185 (27)
Non-Treatment	59 (7)	93 (11)	167 (20)	234 (28)	281 (34)
**Total**	**106 (7)**	**170 (11)**	**324 (22)**	**439 (29)**	**466 (31)**

The enrollment fraction of each race was calculated. Treatment and non-treatment trials were analyzed separately for each race, and the enrollment fraction was highest among Hawaiians for both categories of trials. Compared to Whites, Chinese, Hawaiian, and Japanese patients were enrolled in treatment trials at a significantly higher fraction (p < 0.05). Similarly, for non-treatment trials, Chinese and Hawaiians had a significantly higher enrollment fraction than Whites (Table 3).

**Table 3:** Clinical trial enrollment fraction (%) by race, 2009–2013.

	Race					
**Trial Type**	Chinese	Filipino	Hawaiian	Japanese	White	Total
Treatment	2.5*	1.8	3.2*	2.6*	1.7	2.3
Non-Treatment	3.2*	2.0	3.4*	2.9	2.6	2.8

(*p < 0.05 compared to White).

**Fig. 1:**
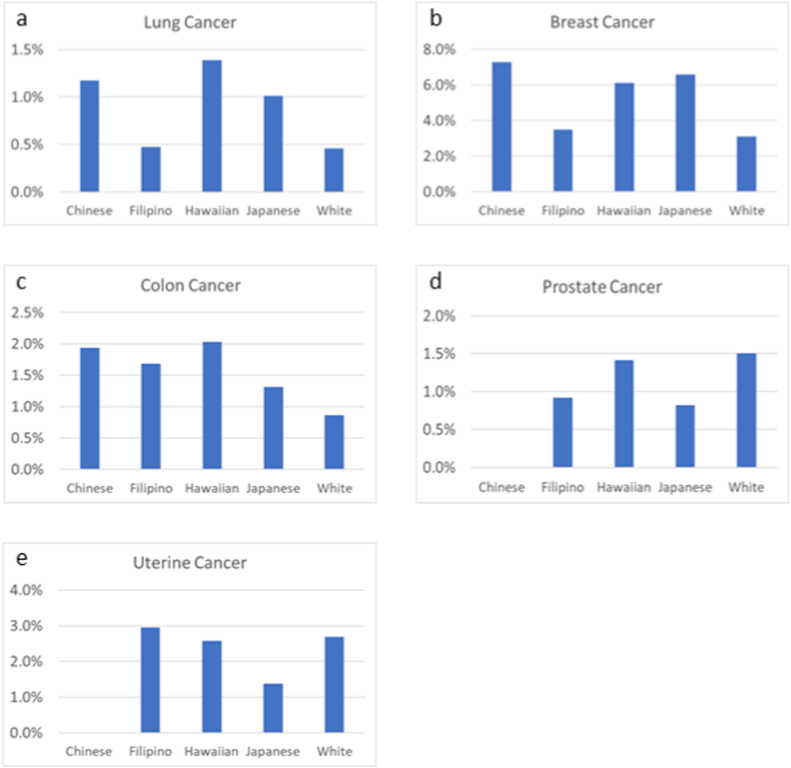
Enrollment fraction for five most common cancers (lung, breast, colon, prostate, and uterine) by race.

Analysis of clinical trial (treatment and non-treatment) participation according to cancer diagnosis was performed for the five most common cancers: lung, breast, colon, prostate, and uterine cancer (Fig. 1a–e). For breast, colon, and lung cancer, all minorities had a higher or similar enrollment fraction than Whites. Conversely, Whites had the highest enrollment fraction of prostate cancer and were second only to Filipinos for uterine cancer.

## 3 Discussion

Our analysis of cancer patients in Hawaii found that Asian and Native Hawaiian minority patients are enrolled at a similar or higher rate than Whites. This is in contrast to other studies that report a significantly lower rate of enrollment of racial minority cancer patients compared to Whites [4,5]. The UHCC experience provides a real-world example of the success of interventions to overcome patient, provider, and health system barriers to minority accrual in cancer clinical trials. These strategies include: (1) involving and hiring minority healthcare providers and research staff, (2) providing minority patients the same clinical trial opportunities as Whites, and (3) obtaining funding to specifically enhance the accrual of minorities.

Patients' lack of trust and confidence in healthcare providers and study investigators are barriers to enrollment of minority patients. A number of groups have recommended the training and hiring of minority healthcare providers and research staff to increase accrual of minority patients to clinical trials [15,[21], [22], [23]]. Minority patients express a higher degree of trust with racially concordant providers [[24], [25], [26], [27], [28]]. In Hawaii, most of the oncology providers and site principal investigators are minorities as are the majority of the research staff, likely contributing to the higher rate of minority accrual at our center.

Healthcare providers perceive that minority patients are poor study candidates [19]. Hawaii is the most diverse state [29] and the first state documented to have fewer than 50% non-Hispanic Whites [30]. This majority-minority population is reflected in the large number of minority cancer patients in the state. In Hawaii, minorities compose the bulk of the patients treated in clinics and hospitals, and they receive the same care and clinical trial opportunities as White patients. Langford et al. showed that racial disparities in the desire to participate and actual participation in clinical trials were eliminated when patients had access to and were offered participation in studies [18].

Insufficient funding to support clinical trial activities in areas that support racial minority patients is a significant barrier to enrollment. UHCC is a Minority/Underserved 10.13039/100000054National Cancer Institute Community Oncology Research Program, and receives federal funding to help achieve its goal of increasing visibility and accessibility of clinical trials to the minorities within its cancer community [20]. The large proportion of minority accrual demonstrates the success of the program. Further targeted federally funded interventions may help to reduce the racial disparity in cancer clinical trial participation.

These strategies have increased the enrollment of Asian and Native Hawaiian cancer patients at least to the level of Whites. UHCC's total clinical trial accrual rate is 5.9%, nearly double the national average. Nevertheless, it is still a small minority of cancer patients being treated in the UHCC network who are participating in studies. It is estimated that 70% of cancer patients are interested in participating in trials [31]. We are collecting data on all patients who are eligible for a cancer trial and documenting specific reasons for declining for those who choose not to participate. These data will be analyzed to develop further strategies to improve accrual for our entire community and specifically Asians and Native Hawaiians. Currently we have focused our efforts to further increase the enrollment of minority patients in clinical trials in two areas: (1) broadened insurance coverage of clinical trial activities and (2) overcoming language barriers to trial participation faced by non-English speakers.

We recognized that the lack of adequate medical insurance is a health systems barrier to appropriate healthcare including clinical trial participation. This is a problem disproportionately faced by racial minorities [32]. UHCC successfully lobbied the state of Hawaii to expand Medicaid coverage of clinical trial activities, and these efforts resulted in the passage of HI SB2324 in 2019. This law requires that clinical trial activities be a covered benefit for patients with Medicaid insurance. Thus, in Hawaii, nearly all minority patients have an insurance plan that provides coverage for clinical trial participation.

UHCC also intends to implement a process to enroll non-English speaking patients in clinical trials. The lack of a feasible mechanism to translate informed consent documents and other study materials such as quality of life measurement tools has likely decreased enrollment of certain minority groups. In particular Filipinos, who represent the largest group of recent immigrants, have a large population of non-English speakers [33]. In 2018, the National Cancer Institute's Central Institutional Review Board approved the use of a translated short form to obtain consent and currently provides the form translated into 43 languages including the Filipino dialect of Tagalog. We are developing a process to utilize the short form across the UHCC network to increase access to clinical trials.

While our study has many strengths, there are some limitations. First, our clinical trials management system, OnCore, does not capture all patients enrolled in clinical trials in the state as about 3% are enrolled at a health maintenance organization that does not participate in the UHCC network [34]. Second, while we present data suggesting an association between the accrual of minority patients and the interventions that we describe, we cannot confirm a direct causal link. Nevertheless, we show that some racial minority groups have a significantly higher enrollment fraction than Whites, and it is likely that these strategies are in part responsible.

In Hawaii, we have demonstrated that Asian and Native Hawaiian minorities participate in clinical trials at rates similar or better compared to Whites. UHCC has implemented a number of strategies to increase clinical trial accrual of minority patients that are likely contributing to this phenomenon. While Hawaii has a unique racial make-up of patients, there are other states with a majority minority (New Mexico, California, Texas, Nevada, and Maryland) and communities with large minority populations that could improve clinical trial accrual through implementation of similar strategies.

## Funding source

None.

## Author contributions

Acoba: conceptualization, formal analysis, writing – original draft, Sumida: writing – reviewing and editing, Berenberg: writing – reviewing and editing.

## Declaration of competing interest

The authors declare that they have no known competing financial interests or personal relationships that could have appeared to influence the work reported in this paper.
